# Elevation of circulating fatty acid-binding protein 4 is independently associated with left ventricular diastolic dysfunction in a general population

**DOI:** 10.1186/s12933-014-0126-7

**Published:** 2014-08-21

**Authors:** Takahiro Fuseya, Masato Furuhashi, Satoshi Yuda, Atsuko Muranaka, Mina Kawamukai, Tomohiro Mita, Shutaro Ishimura, Yuki Watanabe, Kyoko Hoshina, Marenao Tanaka, Kohei Ohno, Hiroshi Akasaka, Hirofumi Ohnishi, Hideaki Yoshida, Shigeyuki Saitoh, Kazuaki Shimamoto, Tetsuji Miura

**Affiliations:** Department of Cardiovascular, Renal and Metabolic Medicine, Sapporo Medical University School of Medicine, S-1, W-16 Chuo-ku, Sapporo, 060-8543 Japan; Department of Clinical Laboratory Medicine, Chuo-ku, Sapporo, 060-8543 Japan; Department of Public Health, Sapporo Medical University School of Medicine, Chuo-ku, Sapporo, 060-8543 Japan; Department of Nursing, Division of Medical and Behavioral Subjects, Sapporo Medical University School of Health Sciences, Chuo-ku, Sapporo, 060-8543 Japan; Sapporo Medical University, S-1, W-16, Chuo-ku, Sapporo, 060-8543 Japan

**Keywords:** Fatty acid-binding protein 4, Adipokine, Left ventricular diastolic dysfunction

## Abstract

**Background:**

Fatty acid-binding protein 4 (FABP4) is expressed in both adipocytes and macrophages. Recent studies have shown secretion of FABP4 from adipocytes and association of elevated serum FABP4 level with obesity, insulin resistance, hypertension, and atherosclerosis. However, little is known about role of FABP4 in cardiac function.

**Methods:**

From the database of the Tanno-Sobetsu Study, data for 190 subjects (male/female: 82/108) who were not treated with any medication and underwent echocardiography in 2011 or 2012 were retrieved for analyses of relationships between serum FABP4 concentration, metabolic markers and parameters of echocardiography.

**Results:**

Serum FABP4 level was positively correlated with age, body mass index (BMI), blood pressure (BP), LDL cholesterol, HOMA-R and mean left ventricular (LV) wall thickness (LVWT, males: r = 0.315, females: r = 0.401, p < 0.01) and was negatively correlated with HDL cholesterol, estimated glomerular filtration rate (eGFR) and peak myocardial velocity during early diastole (e’; males: r = −0.434, females: r = −0.353, p < 0.01), an index of LV diastolic function. However, no significant correlation was found between FABP4 level and LV end-diastolic dimension, LV ejection fraction or LV mass index. There were significant correlations of e’ with age, BMI, BP, eGFR, brain natriuretic peptide (BNP), FABP4, metabolic markers and LVWT. Multivariate regression analysis adjusted by HOMA-R, BMI, eGFR, BNP or LVWT in addition to age, gender and BP revealed that serum FABP4 concentration was independently correlated with e’.

**Conclusions:**

Elevation of circulating FABP4 may contribute to LV diastolic dysfunction in a general population.

## Background

Fatty acid-binding proteins (FABPs) are a group of intracellular lipid chaperones that coordinate lipid responses in cells [1,2]. FABPs are about 14-15-kDa proteins that can reversibly bind hydrophobic ligands, such as saturated and unsaturated long chain fatty acids, with high affinity [1,2]. FABPs have been proposed to facilitate the transport of lipids to specific compartments in the cell. Among FABPs, fatty acid-binding protein 4 (FABP4), known as adipocyte FABP (A-FABP) or aP2, is expressed in adipocytes, macrophages and capillary endothelial cells [1-3]. Emerging evidence indicates that FABP4 acts at the integration between metabolic and inflammatory pathways and plays an important role in the development of insulin resistance and atherosclerosis [4-6]. It has also been demonstrated in experimental models that chemical inhibition of FABP4 could be a therapeutic strategy against insulin resistance, diabetes mellitus, fatty liver disease and atherosclerosis [7].

Adipose tissue is now known to secrete a variety of bioactive molecules called adipokines, such as tumor necrosis factor-α (TNFα), leptin and adiponectin, which are implicated in a wide range of biological phenomena. Interestingly, recent studies have shown that FABP4 is secreted from adipocytes [8,9], though there are no typical signal peptides for secretion in the sequence of FABP4 [1]. It has also been demonstrated that secretion of FABP4 is via a non-classical secretion pathway and that FABP4 acts as an adipokine for the development of hepatic insulin resistance [9]. Furthermore, elevated serum concentration of FABP4 has been shown to be associated with obesity, insulin resistance, hypertension and atherosclerosis [8-12].

Obesity is a risk factor for several kinds of cardiac insults, such as left ventricular (LV) hypertrophy, LV diastolic dysfunction and heart failure with preserved or reduced ejection fraction. It has been suggested that several adipokines provide a direct pathophysiological link between enlarged adipose tissue and obesity-associated cardiac dysfunction [13]. However, little is known about the relationship between circulating FABP4 and cardiac function, especially in a general population. Therefore, we hypothesized that increase in serum FABP4 reflects LV diastolic dysfunction as an early stage of cardiac insults in a general population. To address this hypothesis, we conducted a study to investigate the cross-sectional associations between serum FABP4 concentration and several echocardiographic parameters in subjects who had not regularly taken any medications.

## Methods

### Study population

The Tanno-Sobetsu Study is a study with a population-based cohort design recruiting residents of two rural towns, Tanno and Sobetsu, in Hokkaido and includes annual health examination, pathophysiological assessment of metabolic syndrome and cardiovascular disease, and follow-up survey. A total of 357 female subjects (mean age: 66 ± 13 years) in 2011 and 277 male subjects (mean age: 66 ± 13 years) in 2012 received annual examinations in Sobetsu Town. Female and male participants in 2011 and 2012, respectively, were invited to receive echocardiographic examinations. Subjects who were being treated with any regular medications for diseases were excluded. Other exclusion criteria were atrial fibrillation and conductional abnormalities such as left bundle branch block on electrocardiogram or severe valvular disease and left ventricular hypertrophy (wall thickness >12.5 mm) on echocardiogram. A total of 190 subjects who underwent echocardiography (male/female: 82/108, mean age: 63 ± 13 years) contributed to the present analyses. This study conformed to the principles outlined in the Declaration of Helsinki and was performed with the approval of the Ethical Committee of Sapporo Medical University. Written informed consent was received from all of the subjects.

### Measurements

Medical check-ups were performed between 06:00 h and 09:00 h after an overnight fast. After measuring anthropometric parameters, blood pressure was measured twice consecutively on the upper arm using an automated sphygmomanometer (HEM-907, Omron Co., Kyoto, Japan) with subjects in a seated resting position, and average blood pressure was used for analysis. Body mass index (BMI) was calculated as body weight (in kilograms) divided by the square of body height (in meters). Peripheral venous blood samples were obtained from study subjects after physical examination for complete blood count and biochemical analyses of the serum. The serum samples were analyzed immediately or stored at −80°C until biochemical analyses.

Serum concentration of FABP4 was measured using a commercially available enzyme-linked immunosorbent assay kit for FABP4 (Biovendor R&D, Modrice, Czech Republic). The accuracy, precision and reproducibility of the kit have been described previously [8]. The intra- and inter-assay coefficient variances in the kit were < 5%. Fasting plasma glucose was determined by the glucose oxidase method. Fasting plasma insulin was measured by a radioimmunoassay method (Insulin RIA bead, Dianabot, Tokyo, Japan). Creatinine (Cr) and lipid profiles, including total cholesterol, high-density lipoprotein (HDL) cholesterol and triglycerides, were determined by enzymatic methods. Low-density lipoprotein (LDL) cholesterol level was calculated by the Friedewald equation. Hemoglobin A1c (HbA1c) was determined by a latex coagulation method and was expressed in national glycohemoglobin standardization program (NGSP) scale. Brain natriuretic peptide (BNP) was measured using an assay kit (Shionogi & Co., Osaka, Japan). High-sensitivity C-reactive protein (hsCRP) was measured by a nephelometry method. As an index of renal function, estimated glomerular filtration rate (eGFR) was calculated by an equation for Japanese: eGFR(ml/min/1.73m^2^) = 194 × Cr^(‐ 1.094)^ × age^(‐ 0.287)^ × 0.739 (if female). HOMA-R, an indicator of insulin resistance, was calculated by the previously reported formula: insulin(μU/ml) × glucose(mg/dl)/405.

### Echocardiography

After medical check-ups and collection of urine and blood samples, echocardiographic examinations were performed by three well-experienced echocardiographers who were blinded to clinical data, using Vivid 9 (GE Health Care, Tokyo, Japan) equipped with a 2.5-MHz frequency transducer. Two-dimensional and color tissue Doppler imaging modes were used to obtain images from standard echocardiographic views, including parasternal long-axis and apical four-, three-, and two chamber views at a left lateral decubitus position. Standard parameters in two-dimensional measurements, including LV end-diastolic and end-systolic dimensions (mm) and septal and posterior wall thicknesses at end-diastole (mm), were determined. Mean LV wall thickness (mm) was calculated by the average of septal and posterior wall thicknesses at end-diastole. LV ejection fraction (%) was calculated using biplane modified Simpson’s method. LV mass was calculated according to the recommendations of the American Society of Echocardiography [14] and normalized for body surface area (LV mass index, g/m^2^). Left atrial (LA) dimension (mm) was measured by M-mode echocardiography, and LA volume was measured using biplane Simpson’s method and normalized for body surface area (LA volume index, ml/m^2^) [14]. Each parameter was evaluated by averaging two to three measurements. Transmitral flow velocities were obtained by pulsed wave Doppler echocardiography, positioning a sample volume at the level of a mitral tip in an apical four-chamber view. Mitral flow parameters, including peak velocities during early (E) and late diastole (A) and E-wave deceleration time, were measured, and the E/A ratio was calculated. Tissue velocity curves were obtained from color tissue Doppler imaging. A sample volume was placed at the lateral annulus in the apical four-chamber view, and peak myocardial velocity during early diastole (e’, cm/sec) was measured, and the ratio of mitral to myocardial early diastolic peak velocity (E/e’) was calculated.

### Statistical analysis

Numeric variables are expressed as means ± SD for normal distributions or medians (interquartile ranges) for skewed variables. The distribution of each parameter was tested for its normality using the Shapiro-Wilk W test, and non-normally distributed parameters were logarithmically transformed for comparison and regression analyses. Comparison between two groups was done with an unpaired *t* test. One-way analysis of variance and Tukey-Kramer *post hoc* test were used for detecting significant differences in data between multiple groups. The correlation between two variables was evaluated using Pearson’s correlation coefficient. Multivariate regression analysis was performed to identify independent determinants of e’ using the variables with a significant and non-confounding correlation in simple regression analysis as independent predictors, showing the t-ratio calculated as the ratio of regression coefficient and standard error of regression coefficient and the percentage of variance in the object variables that they explained (R^2^). A p value of less than 0.05 was considered statistically significant. Holm-Bonferroni sequential correction was also performed in multivariate regression analysis. All data were analyzed by using JMP 9 for Macintosh (SAS Institute, Cary, NC).

## Results

Basal characteristics of the study subjects are shown in Table 1. Male subjects were significantly older than the female subjects and they had significantly larger BMI and waist circumference and had higher levels of systolic and diastolic blood pressures, triglycerides, glucose, HbA1c, insulin, HOMA-R and Cr and lower levels of total cholesterol, HDL cholesterol, LDL cholesterol and FABP4 than did the females. No significant difference in eGFR or BNP was found between male and female subjects. In echocardiographic parameters, LA dimension, mean LV wall thickness, LV end-diastolic dimension, LV mass index and E-wave deceleration time were significantly larger in males than in females. On the other hand, LV ejection fraction and E/A ratio were smaller in males than in females. Levels of e’ and E/e’ were comparable between male and female subjects.

**Table 1:** **Characteristics of the studied subjects**

	**Whole**	**Male**	**Female**
**n**	**190**	**82**	**108**
Age (years)	63 ± 13	66 ± 13	60 ± 13*
Body mass index (kg/m^2^)	23.2 ± 3.7	24.4 ± 3.9	22.3 ± 3.4*
Waist circumference (cm)	84 ± 11	88 ± 11	81 ± 10*
Systolic blood pressure (mmHg)	134 ± 21	138 ± 18	130 ± 22*
Diastolic blood pressure (mmHg)	77 ± 11	79 ± 10	75 ± 12†
Biochemical data			
Total cholesterol (mg/dl)	202 ± 33	188 ± 29	212 ± 32*
HDL cholesterol (mg/dl)	68 ± 18	59 ± 17	75 ± 16*
LDL cholesterol (mg/dl)	119 ± 29	108 ± 28	127 ± 28*
Triglycerides (mg/dl)	83 (64–112)	93 (70–132)	77 (57–100)*
Glucose (mg/dl)	92 (87–99)	97 (91–103)	90 (85–96)*
HbA1c (%)	5.5 ± 0.5	5.6 ± 0.5	5.4 ± 0.4*
Insulin (μU/ml)	4.5 (3.4-6.9)	4.9 (3.6-7.6)	4.2 (3.2-6.1)*
HOMA-R	1.02 (0.74-1.63)	1.21(0.85-1.92)	0.94 (0.68-1.37)*
Creatinine (mg/dl)	0.74 ± 0.17	0.87 ± 0.16	0.65 ± 0.11*
eGFR (ml/min/1.73 m^2^)	72 ± 14	71 ± 15	74 ± 14
BNP (pg/ml)	28 (11–31)	14 (9–33)	21 (12–31)
hsCRP (mg/dl)	0.03 (0.02-0.07)	0.04 (0.02-0.08)	0.03 (0.02-0.06)
FABP4 (ng/ml)	12.2 (8.4-16.2)	10.9 (8.1-14.2)	13.3 (9.1-17.0)†
Echocardiographic parameters			
Left atrial dimension (mm)	34 ± 6	36 ± 6	33 ± 6*
Left atrial volume index (ml/m^2^)	27 ± 8	26 ± 8	27 ± 8
Mean LV wall thickness (mm)	8.8 ± 1.2	9.6 ± 0.9	8.2 ± 0.9*
LV end-diastolic dimension (mm)	44 ± 5	45 ± 5	43 ± 4*
LV mass index (g/m^2^)	93 ± 23	102 ± 22	86 ± 21*
LV ejection fraction (%)	67 ± 5	66 ± 6	67 ± 5†
E/A	0.91 (0.75-1.19)	0.79 (0.68-0.94)	0.98 (0.79-1.31)*
E-wave deceleration time (msec)	214 ± 53	223 ± 59	207 ± 47†
e’ (cm/sec)	10.2 ± 3.1	9.7 ± 3.2	10.6 ± 3.0
E/e’	7.0 ± 2.3	6.7 ± 2.3	7.3 ± 2.3

Variables are expressed as number, means ± SD or medians (interquartile ranges). BNP, brain natriuretic peptide; eGFR, estimated glomerular filtration rate; hsCRP, high-sensitivity C-reactive protein; LV, left ventricle. *P < 0.01, †P < 0.05 vs. male.

In analyses of data from all study subjects, serum FABP4 level was positively correlated with age, BMI, systolic and diastolic blood pressures, total cholesterol, LDL cholesterol, triglycerides, insulin, HOMA-R, Cr and hsCRP and was negatively correlated with eGFR (Table 2). Similar correlations between the parameters were observed when male and female subjects were separately analyzed.

**Table 2:** **Simple regression analysis for log FABP4**

	**Whole (n = 190)**		**Male (n = 82)**		**Female (n = 108)**	
	**r**	**p**	**r**	**p**	**r**	**p**
Age	0.225	0.002	0.193	0.083	0.314	0.001
Body mass index	0.500	<0.001	0.580	<0.001	0.564	<0.001
Waist circumference	0.518	<0.001	0.642	<0.001	0.518	<0.001
Systolic blood pressure	0.272	<0.001	0.283	0.010	0.329	0.001
Diastolic blood pressure	0.268	<0.001	0.255	0.021	0.329	0.001
Biochemical data						
Total cholesterol	0.213	0.003	0.008	0.943	0.282	0.003
LDL cholesterol	0.268	<0.001	0.058	0.606	0.365	<0.001
HDL cholesterol	−0.107	0.143	−0.125	0.263	−0.238	0.013
log Triglycerides	0.157	0.031	0.097	0.385	0.282	0.003
log Glucose	0.137	0.060	0.081	0.467	0.277	0.004
HbA1c	0.046	0.532	0.044	0.695	0.107	0.272
log Insulin	0.388	<0.001	0.380	0.001	0.480	<0.001
log HOMA-R	0.386	<0.001	0.384	<0.001	0.489	<0.001
Creatinine	0.192	0.008	0.448	<0.001	0.305	0.001
eGFR	−0.350	<0.001	−0.397	<0.001	−0.350	<0.001
log BNP	0.126	0.085	0.076	0.500	0.156	0.108
log hsCRP	0.233	0.001	0.312	0.005	0.218	0.024
Echocardiographic parameters						
Left atrial dimension	0.251	0.001	0.354	0.001	0.290	0.002
Left atrial volume index	0.231	0.002	0.226	0.050	0.225	0.020
Mean LV wall thickness	0.195	0.007	0.315	0.004	0.401	<0.001
LV end-diastolic dimension	−0.042	0.562	0.069	0.541	−0.082	0.399
LV mass index	0.039	0.596	0.085	0.448	0.105	0.281
LV ejection fraction	0.113	0.121	0.219	0.048	−0.023	0.812
log E/A	−0.178	0.016	−0.173	0.131	−0.279	0.004
E-wave deceleration time	0.006	0.934	−0.013	0.906	0.066	0.497
e’	−0.360	<0.001	−0.434	<0.001	−0.353	<0.001
E/e’	0.353	<0.001	0.361	0.001	0.327	0.001

BNP, brain natriuretic peptide; eGFR, estimated glomerular filtration rate; hsCRP, high-sensitivity C-reactive protein; LV, left ventricle.

**Figure 1:**
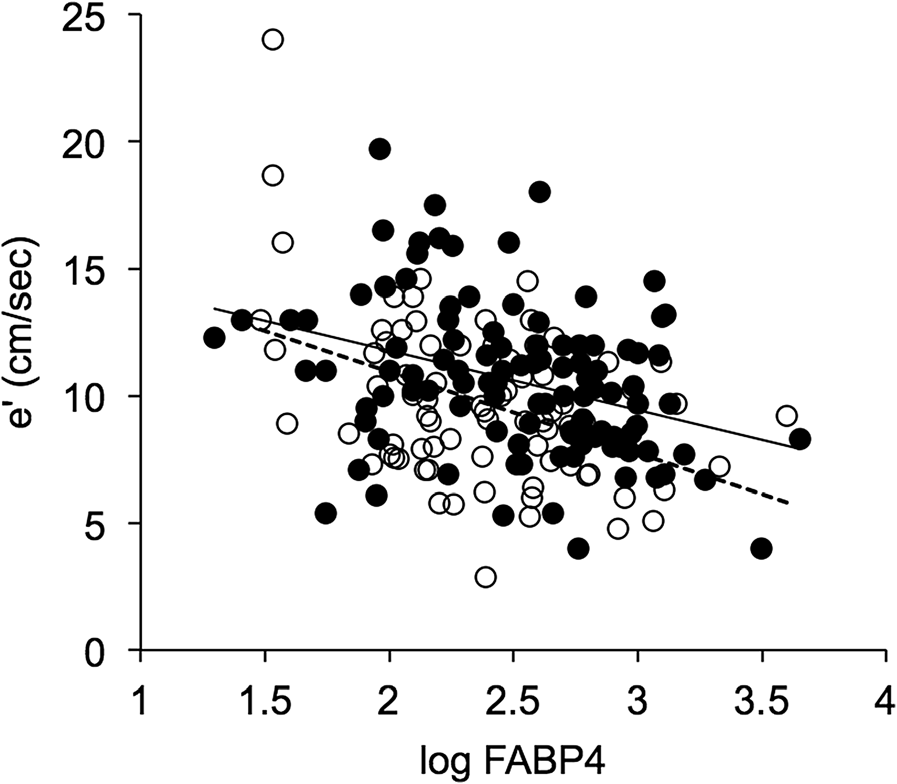
**Association of serum FABP4 level with LV diastolic dysfunction.** Peak myocardial velocity during early diastole (e’), an index of LV diastolic function, was plotted against log FABP4 for each subject. There was a significant correlation between the two parameters (males: r = −0.434, females: r = −0.353, p < 0.001). Open circles: males (n = 82), closed circles: females (n = 108). Solid regression line: males, broken regression line: females.

Regarding echocardiographic parameters, FABP4 concentration was positively correlated with LA dimension, LA volume index and mean LV wall thickness (males: r = 0.315, females: r = 0.401, p < 0.01), though correlation with FABP4 was not significant for LV end-diastolic dimension or LV mass index. FABP4 level was positively correlated with E/e’ and negatively correlated with e’ (Figure 1; males: r = −0.434, females: r = −0.353, p < 0.001), an index of LV diastolic function, and E/A ratio (Table 2), whereas LV ejection fraction was not correlated with FABP4 level. Among echocardiographic parameters, e’ was positively correlated with LV ejection fraction and E/A ratio and was negatively correlated with LA dimension, LA volume index, mean LV wall thickness, LV mass index, E-wave deceleration time and E/e’ (Table 3). Of extra-cardiac parameters, age, BMI, waist circumference, systolic and diastolic blood pressures and biochemical markers, including eGFR, BNP, hsCRP and FABP4, were found to be significantly correlated with e’ (Table 3).

**Table 3:** **Simple regression analysis for LV diastolic function**

	**e’**	
	**t**	**p**
Age	−0.713	< 0.001
Body mass index	−0.200	0.006
Waist circumference	−0.324	< 0.001
Systolic blood pressure	−0.465	< 0.001
Diastolic blood pressure	−0.233	0.001
Biochemical data		
Total cholesterol	−0.071	0.334
LDL cholesterol	−0.140	0.054
HDL cholesterol	0.175	0.016
log Triglycerides	−0.148	0.042
log Glucose	−0.195	0.007
HbA1c	−0.210	0.004
log Insulin	−0.156	0.032
log HOMA-R	−0.187	0.010
Creatinine	−0.231	0.001
eGFR	0.376	< 0.001
log BNP	−0.394	< 0.001
log hsCRP	−0.201	0.006
log FABP4	−0.360	< 0.001
Echocardiographic parameters		
Left atrial dimension	−0.289	< 0.001
Left atrial volume index	−0.233	0.002
Mean LV wall thickness	−0.370	< 0.001
LV end-diastolic dimension	0.024	0.739
LV mass index	−0.294	< 0.001
LV ejection fraction	0.148	0.043
log E/A	0.644	< 0.001
E-wave deceleration time	−0.205	0.005
E/e’	−0.580	< 0.001

BNP, brain natriuretic peptide; eGFR, estimated glomerular filtration rate; hsCRP, high-sensitivity C-reactive protein; LV, left ventricle.

Multivariate regression analysis was performed to identify independent determinants of e’ using systolic blood pressure, the most strongly correlated factor among anthropometric and biochemical parameters (r = −0.465, p < 0.001), in addition to age and gender (Model 1) and showed that serum FABP4 concentration was independently correlated with e’ (Table 4). Next, the variables with a significant and non-confounding correlation in simple regression analysis were additionally chosen as possible independent predictors in Model 2 ~ 6: a marker of adiposity (BMI, Model 2), glucose and insulin metabolism (HOMA-R, Model 3), renal function (eGFR, Model 4), cardiac damage (BNP, Model 5) or cardiac morphology (LV wall thickness, Model 6). When the each parameter was additionally incorporated into the adjustment, FABP4 remained as an independent predictor of e’ in Model 2 ~ 6 (Table 4), although the independent correlation in Model 2 was cancelled after Holm-Bonferroni sequential correction. Additional multivariate regression analysis using all of the used parameters in Model 1 ~ 6, including age, gender, systolic blood pressure, BMI, HOMA-R, eGFR, BNP, mean LV wall thickness and FABP4, showed that FABP4 level (t = −2.36, p = 0.020) was independently correlated with e’ after adjustment of other variables (overall R^2^ = 0.563).

**Table 4:** **Multivariate regression analysis for LV diastolic function**

	**e’**			**e’**	
**Model**	**t**	**p**	**Model**	**t**	**p**
**Model 1**			**Model 4**		
Age	−10.64	< 0.001	Age	−10.27	< 0.001
Gender (Male)	−0.31	0.755	Gender (Male)	−0.39	0.695
Systolic blood pressure	−1.83	0.069	Systolic blood pressure	−1.88	0.062
log FABP4	−3.69	< 0.001	eGFR	−1.34	0.181
	R^2^ = 0.558		log FABP4	−3.93	< 0.001
				R^2^ = 0.562	
**Model 2**			**Model 5**		
Age	−10.64	< 0.001	Age	−9.11	< 0.001
Gender (Male)	0.27	0.788	Gender (Male)	−0.33	0.744
Systolic blood pressure	−1.57	0.118	Systolic blood pressure	−1.80	0.073
Body mass index	−1.28	0.203	log BNP	−0.33	0.742
log FABP4	−2.29	0.023	log FABP4	−3.70	< 0.001
	R^2^ = 0.562			R^2^ = 0.556	
**Model 3**			**Model 6**		
Age	−10.45	< 0.001	Age	−10.34	< 0.001
Gender (Male)	0.09	0.925	Gender (Male)	0.17	0.866
Systolic blood pressure	−1.74	0.083	Systolic blood pressure	−1.62	0.107
log HOMA-R	−0.89	0.376	Mean LV wall thickness	−0.67	0.503
log FABP4	−3.01	0.003	log FABP4	−3.40	< 0.001
	R^2^ = 0.560			R^2^ = 0.559	

BNP, brain natriuretic peptide; eGFR, estimated glomerular filtration rate; LV, left ventricle.

**Figure 2:**
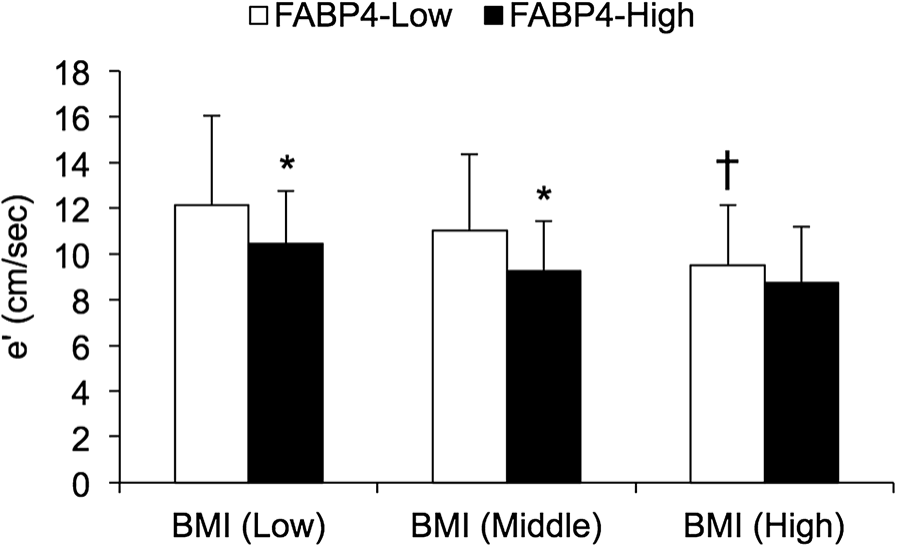
**Comparison of LV diastolic function between subjects with low and high levels of FABP4 in tertiles of BMI.** Peak myocardial velocity during early diastole (e’), an index of LV diastolic function, was compared between subjects with low and high levels of FABP4 divided by median value of FABP4 levels (FABP4-Low and FABP4-High, respectively) in tertiles of body mass index (BMI). Tertile of BMI consists of low (<21.4 kg/m^2^; n = 63), middle (≥21.4 and <24.4 kg/m^2^; n = 63), and high (≥24.4 kg/m^2^; n = 64) groups. *P < 0.05 vs. FABP4-Low. †P < 0.05 vs. FABP4-Low in BMI (Low).

In low and middle tertiles of BMI, e’ in a group with low levels of FABP4 (FABP4-Low) was significantly higher than that in a group with high levels of FABP4 (FABP4-High) (Figure 2). Furthermore, there was no significant difference in e’ between the FABP4-Low and FABP4-High groups in high tertile of BMI, but the FABP4-Low group in high tertile of BMI had significantly lower e’ than did that in low tertile of BMI.

## Discussion

The salient finding in the present study was that FABP4 was independently and negatively correlated with e’, which reflects LV relaxation and is known as one of the most sensitive indexes of LV diastolic function in a healthy population [14]. LV diastolic dysfunction often precedes LV systolic dysfunction in heart diseases, and moderate diastolic dysfunction alone potentially induces heart failure, which is referred to as heart failure with preserved ejection fraction (HFpEF) [15]. A recent study in which data from the Framingham cohort study were analyzed showed that age, diabetes mellitus, BMI, smoking and atrial fibrillation were predictors of HFpEF [16]. It is notable that the correlation of FABP4 level with e’ was independent of age, BMI, HOMA-R and LV wall thickness (Table 4). These results suggest that serum FABP4 is a novel marker of LV diastolic dysfunction and potentially a predictor of HFpEF.

Previous studies using animal models indicated that FABP4 plays a significant role in several aspects of metabolic syndrome, including insulin resistance, type 2 diabetes and atherosclerosis, through its action at the interface of metabolic and inflammatory pathways in adipocytes and macrophages [1,2,4-6]. Epicardial fat has been reported to directly influence cardiac function because of the absence of a fibrous fascial layer between fat and the underlying myocardium [17,18]. FABP4 mRNA expression in epicardial adipose tissue was recently reported to be profoundly increased compared with its expression in paraaortic adipose tissue in patients with metabolic syndrome [19]. Furthermore, it has recently been reported that exogenous FABP4 acutely suppresses shortening amplitude in cardiomyocytes by attenuating intracellular systolic peak Ca^2+^ level in a dose-dependent manner [20] and impairs the insulin-dependent nitric oxide pathway in vascular endothelial cells [21]. Therefore, it is possible that either FABP4 secreted from epicardial fat tissue or circulating FABP4 released from subcutaneous and/or visceral adipose tissue or from macrophages may directly modulate cardiac function. In the heart, FABP3 known as heart-type FABP (H-FABP) is abundant and is rapidly released from cells into the circulation after onset of cardiomyocyte damage. Serum concentration of FABP3 has been characterized as an early biochemical marker of acute myocardial infarction and a sensitive marker of ongoing myocardial damage in patients with heart failure [22,23]. Impact of circulating FABP3 is apparently different from that of FABP4.

Inflammation is an important factor in the pathogenesis and progression of heart failure. It has been shown that increased inflammatory cytokines produced by mononuclear cells including macrophages and/or damaged myocardium impaired myocardial function by inducing apoptosis, necrosis and hypertrophic response in cardiomyocytes [24]. In the Framingham Heart Study, increased inflammatory markers, such as CRP, interleukin-6 and TNFα levels, were able to identify asymptomatic older subjects in the community who were at high risk for the future development of heart failure [25]. In the present study, FABP4 was positively correlated with hsCRP, being consistent with the results of several previous studies [10,12]. The macrophage is a critical site of FABP4 action, and macrophage-specific FABP4 deficiency leads to reduced activation of nuclear factor κ B (NF-κB) and c-Jun N-terminal kinase (JNK), resulting in reduced production of a cluster of inflammatory cytokines [5]. Conversely, several inflammatory stimuli have been shown to cause significantly increased expression of FABP4 in macrophages [5]. Local inflammation mediated by FABP4 in macrophages of the heart may participate in mediating cardiac dysfunction.

Up-regulation of FABP4 expression and other adipokines in heart failure has been demonstrated in recent studies [26-28], indicating complex neurohormonal and metabolic abnormalities associated with heart failure. Of note, up-regulation of inflammatory cytokines, catecholamines and natriuretic peptides in heart failure is known to mediate increased lipolysis and insulin resistance [29]. It has been reported that lipolysis is mediated in part through the interaction of FABP4 with hormone-sensitive lipase in adipocytes [30]. A recent study also showed that FABP4 is secreted from adipocytes in a non-classical secretion pathway in relation to lipolysis [9]. Although most of the recruited subjects in the present study were considered to be healthy, relatively high level of lipolytic stimuli, such as inflammatory cytokines, catecholamines and natriuretic peptides, in asymptomatic cardiac dysfunction may increase serum FABP4 concentration.

Circulating FABP4 level was associated with increased LV mass in overweight and obese women [31] and in patients with obstructive sleep apnea syndrome [32]. Recent studies also showed an independent correlation of elevated serum FABP4 with NT-proBNP in heart failure patients [33] or deterioration of LV systolic function in non-obese patients hospitalized for acutely decompensated heart failure [26] and in patients with coronary artery disease [34]. In contrast, there was no significant association between FABP4 level and concurrent [32] or subsequently developed [27] systolic dysfunction in subjects without obvious cardiac disease. In the present study using apparently healthy subjects with no medication, serum FABP4 level was weakly correlated with mean LV wall thickness but with LV mass index or LV ejection fraction. These findings suggest only a marginal contribution of FABP4 to development of the early phase of LV hypertrophy and systolic dysfunction.

Similar to our results, a very recent study by Baessler et al. [35] demonstrated that FABP4 level was independently correlated with e’ after adjustment of age, sex and adiposity in 96 obese subjects and 24 healthy normal weight control subjects, although the association of FABP4 levels with LV diastolic dysfunction was mainly observed in obese subjects with metabolic complications but not in metabolically healthy obese subjects. However, LV diastolic dysfunction in the previous study was defined by combination of several parameters, such as e’, E/e’, E/A, E-wave deceleration time and left atrial dimension. This definition may affect the results. Of note, we showed that FABP4 level was an independent predictor of e’, which is known as an index of LV relaxation and one of the most sensitive indicator of LV diastolic function compared with other indices, especially in a healthy population [14].

A genetic variant at the FABP4 locus associated with decreased FABP4 expression in adipose tissue has been reported to reduce the risk of cardiovascular disease in a population study [36]. We and others previously showed that serum FABP4 level predicts long-term cardiovascular events [37-39]. Furthermore, a large-scale prospective study showed that concentration of FABP4 predicted the risk of heart failure during a median follow-up of 10.7 years [27]. Accumulating evidence of a causative role of FABP4 in cardiac dysfunction would prove that FABP4 is a novel target for prevention of heart failure.

Since FABP4 is a low-molecular-weight protein and freely filtered at the glomerulus, a decrease in glomerular function was shown to result in an elevation of FABP4 concentration [37]. In the present study, FABP4 was negatively correlated with eGFR but remained as an independent predictor of LV diastolic dysfunction even after adjusting for renal function. Besides eGFR, multivariate regression analysis demonstrated that the association of FABP4 level with LV diastolic dysfunction was independent of blood pressure, LV wall thickness and BNP, a well-known predictor of cardiac damage.

The present study has some limitations. Since it has been reported that several drugs, including statin, angiotensin II receptor blocker and peroxisome proliferator-activated receptor γ agonist, affect FABP4 concentrations [40-42], we excluded subjects who had been treated with any drugs in the present study. Therefore, only a small number of subjects could be enrolled, and the statistical power was not large. Another limitation of this study is its cross-sectional design. Prospective longitudinal studies using larger numbers of subjects with no medication are necessary for determining whether FABP4 level is indeed a major determinant of subsequent development of cardiac dysfunction. In addition, the results of our study rely on correlation analyses. A direct relationship between FABP4 level and progression of LV diastolic dysfunction remains unclear. This issue warrants further investigation using an interventional approach.

## Conclusions

The present study is the first study to show an independent association of serum FABP4 level with LV diastolic dysfunction in a general population. The increase in serum FABP4 concentration might precede development of the early phase of cardiac dysfunction. Whether FABP4 can serve as a biomarker for early diagnosis of high-risk individuals with heart disease and a potential therapeutic target for cardiac dysfunction warrants further investigation.
